# The use of HE4, CA125 and CA72-4 biomarkers for differential diagnosis between ovarian endometrioma and epithelial ovarian cancer

**DOI:** 10.1186/1757-2215-6-44

**Published:** 2013-07-01

**Authors:** Emanuela Anastasi, Teresa Granato, Renato Falzarano, Paola Storelli, Adele Ticino, Luigi Frati, Pierluigi Benedetti Panici, Maria Grazia Porpora

**Affiliations:** 1 Department of Molecular Medicine, “Sapienza” University of Rome, Policlinico Umberto I, Viale Regina Elena 324, Rome 00161, Italy; 2 CNR-IBPM, National Research Council, Piazzale Aldo Moro 7, Rome 00185, Italy; 3 Department of Gynaecology, Obstetrics and Urology, ”Sapienza” University of Rome, Policlinico Umberto I, Viale del Policlinico 155, Rome 00161, Italy

**Keywords:** CA125, HE4, CA72-4, Tumor markers, Ovarian endometrioma, Adnexal mass, Ovarian cancer

## Abstract

**Background:**

Endometriosis is frequently associated with high levels of CA125. This marker is therefore not useful for discriminating ovarian endometrioma from ovarian malignancy. The aim of this study was to establish a panel of complementary biomarkers that could be helpful in the differential diagnosis between ovarian endometriosis or other ovarian benign masses and ovarian cancer.

**Methods:**

Blood samples from 50 healthy women, 17 patients with benign ovarian tumors, 57 patients with ovarian endometrioma and 39 patients with ovarian cancer were analyzed and serum values were measured for the following biomarkers: CA125, HE4 and CA72-4.

**Results:**

Serum CA125 concentration was elevated in both patients with ovarian endometriosis and ovarian cancer but not in patients with other benign ovarian masses. HE4 was never increased in patients with endometriosis or benign masses whereas it was significantly higher in all patients with ovarian cancer (p < 0.05). A marked difference in CA72-4 values was observed between women with ovarian cancer (67%) and those with endometriosis (p < 0.05).

**Conclusions:**

The results of the study suggest that HE4 and CA72-4 determination is the best approach to confirm the benign nature of ovarian endometrioma in women with high CA125 levels.

## Background

Endometriosis is a common chronic disease, present in 5-10% of women in reproductive age [1]. The disease, characterized by the presence and growth of endometrial tissue outside the uterine cavity, is often associated with infertility and pelvic pain and it tends to recur [2,5]. Endometriosis can be diagnosed by clinical and ultrasound examinations (US) but the most accurate procedure to confirm the diagnosis is laparoscopy that allows visualization of lesions and histological confirmation [6]*.*

Endometriosis is a benign disease but it shares several characteristics with invasive cancer. Cancer antigen 125 (CA125) measurement is an important component in the work-up of a woman with an adnexal mass [7]. However, CA125 is characterized by a low diagnostic specificity, as abnormally high concentrations can be found in malignancies of different origin including non-ovarian gynecological cancer, such as endometrial, pancreatic, lung, breast and colorectal cancer [8]. In patients with endometriosis CA125 levels can be high. In fact, CA125 is the most extensively investigated and used peripheral biomarker of endometriosis [9]. In addition, elevated serum levels of CA125 are associated with non-gynecological diseases such as tuberculosis, liver cirrhosis and also in physiological conditions such as pregnancy or different phases of the menstrual cycle [10,11]. Thus, CA125 has a limited role in the differential diagnosis between endometriosis and ovarian cancer due to the lack of specificity [12]. Recently, the role of surgery for the treatment of ovarian endometriosis in women with pregnancy desire has been criticized because of the fear of ovarian health tissue damage [6,13,14]. In selected cases, particularly in women undergoing assisted reproductive techniques, it is mandatory rule out an ovarian malignancy before ovarian stimulation and embryo-transfer [15]. Misdiagnosed ovarian cancer has been found in women with suspected ovarian endometriosis [16,17].

Therefore identification of non-invasive and accessible markers of epithelial ovarian carcinoma (EOC) is valuable. For this reason serum tumor markers are being increasingly used for the differential diagnosis of adnexal masses.

Recently, the human epididymis protein 4 (HE4) has proved to be a promising marker for epithelial ovarian cancer with higher specificity and sensitivity than CA125 in distinguishing malignant from benign pelvic masses [18,19]. Particularly, measuring both HE4 and CA125 serum concentrations increases the accuracy of ovarian cancer diagnosis and provides valuable information for discriminating ovarian tumors from ovarian endometriotic cysts [20,21] or other gynecologic conditions [8]. However, an increased HE4 levels have been observed also in other types of cancer, i.e. lung adenocarcinoma [22] and in patients with impaired renal function [23].

Other biomarkers have been studied for clinical application in EOC. Among these, cancer antigen 72–4 (CA72-4), a glycoprotein, which increases in gastric, colon, breast and ovarian adenocarcinomas, may be employed alone or in combination with CA125 and HE4. CA72-4 is less sensitive than CA125 for EOC, but it is not influenced by pregnancy or the phase of menstrual cycle [24,25].

The aim of this study was to evaluate the role of those biomarkers, which are usually elevated in patients with EOC, that could be useful to confirm the nature of ovarian cystic endometriosis or other benign ovarian masses.

## Methods

From June 2012 to February 2013, 115 consecutive Italian women (mean age: 35 years, range: 22–82) referred to the Department of Gynecology, Obstetrics and Urology at the University of Rome ”Sapienza” for the presence of an adnexal mass detected at clinical and ultrasound examinations were enrolled in the study. Control group consisted of 50 healthy women (mean age: 30 years, range: 21–57) with clinical and US outcome negative for ovarian masses.

Exclusion criteria included current hormonal therapy, pregnancy, chronic diseases or other types of cancer. Two patients were excluded from the study because they were at the beginning of pregnancy. The study was part of a study protocol approved by local Ethics Committees. All patients signed written informed consent to the study. At enrolment, medical history was collected and peripheral blood samples were drawn from all women and immediately sent to the laboratory for analysis of tumor markers. All groups underwent complete physical and gynecological examination and transvaginal ultrasound (TVUS) with color Doppler imaging.

Women diagnosed with a pelvic mass subsequently underwent surgery. Disease was confirmed by histopathological examination. The women were divided into the following 4 Groups:

*Group 1:* 50 healthy women (mean age: 30 years, range: 21–57) with clinical and US examinations negative for ovarian masses.

*Group 2:* 17 patients with benign ovarian tumors (mean age: 40 years, range: 20–74) with clinical and instrumental diagnosis of benign adnexal disease. Mean diameter of cysts was 57 ± 30.6 mm (range 20–110). Histopathology confirmed mature teratoma in 6 patients (35%) and simple serous cyst in 11 patients (65%).

*Group 3:* 57 patients with ovarian endometrioma (mean age: 36 years, range: 23–48). Diagnosis of endometriosis was achieved on the basis of medical history, clinical and pelvic transabdominal and/or transvaginal US examinations. Patients with indeterminate findings underwent pelvic magnetic resonance imaging (MRI) to confirm suspected endometriosis using the previously described technique [26,27]. At laparoscopy, the disease was staged according to the rASRM classification [28]. Mean diameter of endometriomas was 33 ± 18.9 mm (range 10–80). One patient had both endometriosis and an ovarian dermoid cyst.

*Group 4*: 39 patients with ovarian carcinoma (mean age: 64 years, range: 28–91). Histology confirmed the diagnosis and staging was made according to the International Federation of Gynecology and Obstetrics (FIGO)[29].

Population characteristics are summarized in Table 1.

**Table 1:** Patient population characteristics

**Diagnosis**	**Mean age**	**n**	**Classification**			
**Healthy**	30	50				
**Ovarian Cyst**	40	17				
*Serous cyst*		*11*				
*Dermoid cyst*		*6*				
			**ASRM STAGE**			
			**I**	**II**	**III**	**IV**
**Endometriosis**	36	57	-	6	26	25
			**FIGO STAGE**			
			**I**	**II**	**III**	**IV**
**EOC**	64	39	3	2	4	30

### Sample preparation

All sera were acquired following a standard collection protocol. Briefly, samples were collected in a Red Top Vacutainer, clotted 60–90 min and centrifuged for 10 min at 1300 × g. The serum fractions were aliquoted and stored at −80°C until analysis.

### CA125 determination

Lumipulse® G1200 CA125II is an assay system for the quantitative measurement of CA125 in specimens based on chemiluminescent enzyme immunoassay technology (CLEIA) by a two-step sandwich method (Innogenetics-Fujirebio, Belgium-Japan). This assay makes use of solid phase and ALP-labeled monoclonal antibodies (OC125 and M11 respectively).

CA125 in specimens specifically binds to anti-CA125 monoclonal antibody immobilized on the particles forming antigen-antibody immunocomplexes. The particles are then washed and rinsed in order to remove unbound materials. Alkaline phosphatase (ALP)-labeled anti-CA125 monoclonal antibody specifically binds to CA125 of the immunocomplexes. After a second wash, substrate solution is added. AMPPD contained in the substrate solution is dephosporylated by the catalysis of ALP indirectly conjugated to the particles. A luminescent signal is generated by the cleavage reaction of dephosphorylated 3-(2′-spiroadamantyl)-4-methoxy-4-(3″-phosphoryloxy)-phenyl-1,2-dioxetane (AMPPD) and reflects the amount of CA125 in the sample. Normal levels of CA125 were considered less than 35 U/mL.

### HE4 determination

HE4 levels were determined using the HE4 enzymatic immunoassay (EIA)(Fujirebio Diagnostics). The HE4 EIA is a solid phase, non competitive immunoassay based upon the direct ”sandwich” technique using two monoclonal antibodies, 2H5 and 3D8, directed against two epitopes in the C-WFDC domain of HE4. Controls or patient serum samples and standards were incubated with biotinylated anti-HE4 monoclonal antibody 2H5 aliquots in streptavidin coatedmicrostrips. HE4 present in standards or serum samples was adsorbed to the streptavidin coatedmicrostrips by the biotinylated anti-HE4 monoclonal antibody during the incubation period. The strips were then washed and incubated with horseradish peroxidase (HRP) labeled anti-HE4 monoclonal antibody 3D8. After washing, buffered substrate/chromogen reagent was added to each well and the enzyme reaction was allowed to proceed. During the enzyme reaction a blue color developed if the antigen was present. The intensity of the color was directly proportional to the amount of HE4 present in the samples. According to the manufacturer’s indications, normal values of HE4 were considered less than 150 pmol/L.

#### CA72-4 assay

CA72-4 was detected utilizing a solid phase two-site immunoradiometric ELSA- CA72-4 assay (Cisbio Bioassays, France). Two monoclonal antibodies were prepared against sterically remote antigenic sites on the TAG 72 molecule: the first was coated on the ELSA solid phase, the second, radiolabeled with iodine 125, was used as tracer. TAG 72 molecules present in the standards or the samples to be tested were “sandwiched” between the two antibodies. Following the formation of the coated antibody/antigen/ antibody sandwich, the unbound tracer was easily removed by a washing step. The radioactivity bound to the Elsa was proportional to the concentration of TAG 72 present in the sample. Normal levels of CA72-4 were considered to be less than 3.8 U/mL.

#### Statistical analysis

Women were stratified by disease in four groups. In each group, the median, range, mean, SD for serum CA125, HE4and CA72-4 levels were determined. Mann–Whitney test was used to assess difference in distributions of tumor markers between different patient populations. Log base 10–transformed whisker-box plots were generated for each marker by disease group. The diagnostic performance of the markers was also expressed as sensitivity, specificity, positive predictive values (PPV) and negative predictive values (NPV) using the following cut-off values: 35 U/mL for CA125, 150 pmol/L for HE4, 3.8 U/mL for CA72-4. Receiver operator characteristic (ROC) curves were constructed and the areas under the curve (AUC) with binomial exact 95% confidence intervals (95% CI) were calculated. The method described by DeLong et al. was used to calculate the difference between two AUCs [30]. For all statistical comparisons, a level of P < 0.05 was accepted as statistically significant. All statistical analyses were performed using MedCalc v.12.2.1.0.

## Results

### Biomarker distribution

CA125, HE4 and CA72-4 serum marker levels were evaluated in all groups (163 women). Results expressed as mean, median and ranges are shown in Table 2.

**Table 2:** Serum markers for each group

	**n**	**Group 1**	**Group 2**	**Group 3**	**Group 4**
		**50**	**17**	**57**	**39**
**CA125**	**Mean**	16.8	19.9	46.1	1976.3
U/mL	**SD**	8.6	20.5	34	7390.4
	**Median(range)**	14 (9–47)	13 (9–97)	38 (8–167)^a^	480 (8–46210)^a,b^
**HE4**	**Mean**	48.6	60.6	53.8	508.3
pmol/L	**SD**	13.6	26.5	15.3	301.5
	**Median(range)**	47 (24–103)	58 (30–125)	53 (26–98)	426 (48–850)^a,b^
**CA72-4**	**Mean**	2.8	2.7	3	39.8
U/mL	**SD**	0.45	0.32	0.98	45.1
	**Median(range)**	2.7 (2.1 - 4)	2.8 (2.1 - 3.3)	2.7 (1.8 -6.2)	7 (1–112)^a,b^

^a^p-value < 0.05 vs Group 1 (healthy); ^b^p-value < 0.05 vs Group 2 (Benign) and Group 3 (endometriosis); Group 4 (ovarian cancer).

**Figure 1:**
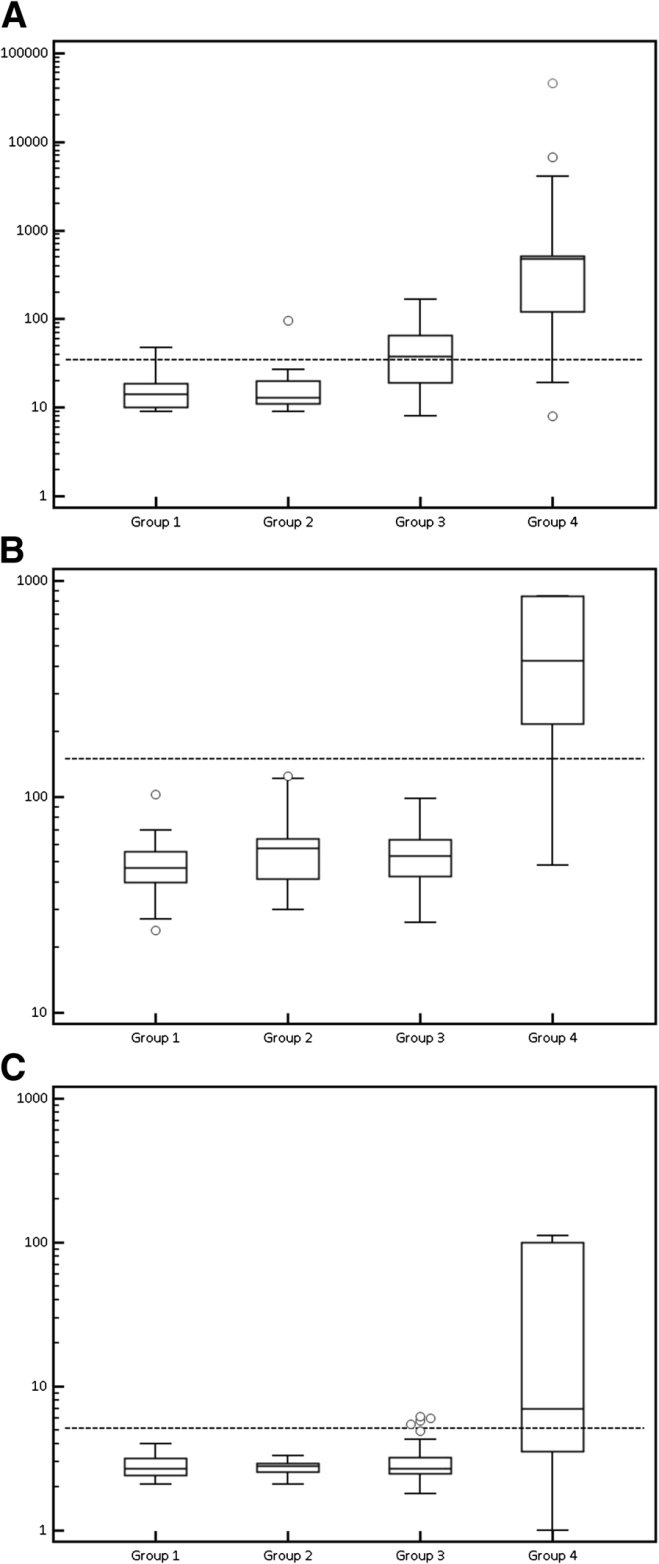
**Box and whisker plots representing median levels and the interquartile range (box) of (A) CA125, (B) HE4 and (C) CA72-4 for each studied group.** The dashed horizontal line represents the cut-off level for each marker (CA125 = 35 U/mL; HE4 = 150 pmol/L; CA72-4 = 3.8 U/mL). The y axis is a logarithmic scale. Group 1 = Healthy women; Group 2 = Ovarian cyst; Group 3 = Endometriosis; Group 4 = Epithelial Ovarian Cancer.

All markers showed significant difference between Group 1 and Group 2 and Group 4 (p < 0.05). HE4 and CA72-4 were significantly higher in Group 4 than in all the other groups (p < 0.05). CA125 was significantly higher in Group 3 and Group 4 than in Group 1 and Group 2 (p < 0.05). The distribution of marker levels for each studied group is shown in Figure 1.

### Tumor marker sensitivity and specificity in malignant and benign disease

In Group 4, HE4 was increased in 89.7% (35/39) of cases, in this group CA72-4 was elevated in 67% (26/39) of cases, while CA125 was positive in 92% (36/39) of patients.

In Group 3, CA125 was elevated in 56.1% (32/57) of cases and a slight but not statistically significant increase of CA72-4 was observed in 7% (4/57) of patients.

HE4 correctly discriminated malignant from benign disease (Group 2 and Group 3 *vs* Group 4) with a sensitivity and specificity of 87% and 100%, respectively. PPV and NPV of HE4 were 100% and 96% respectively.

In patients with malignancy, CA125 showed a significantly higher sensitivity than CA72-4 (90% *vs* 67%, p < 0.001), but a lower specificity than CA72-4 (70% *vs* 96%, p < 0.001). PPV was 51% and 84%, and NPV was 95% and 89% for CA125 and CA72-4, respectively (Table 3).

**Table 3:** **Sensitivity, specificity, PPV and NPV of controls and malignant *vs *benign cases for each marker**

	**CA125**	**HE4**	**CA72-4**
**Sensitivity %**	90	87	67
**Specificity %**	70	100	96
**PPV**	51	100	84
**NPV**	95	96	89

Cut-off levels: CA125 = 35 U/mL; HE4 = 150 pmol/L; CA72-4 =3.8 U/mL.

### Diagnostic accuracy

**Figure 2:**
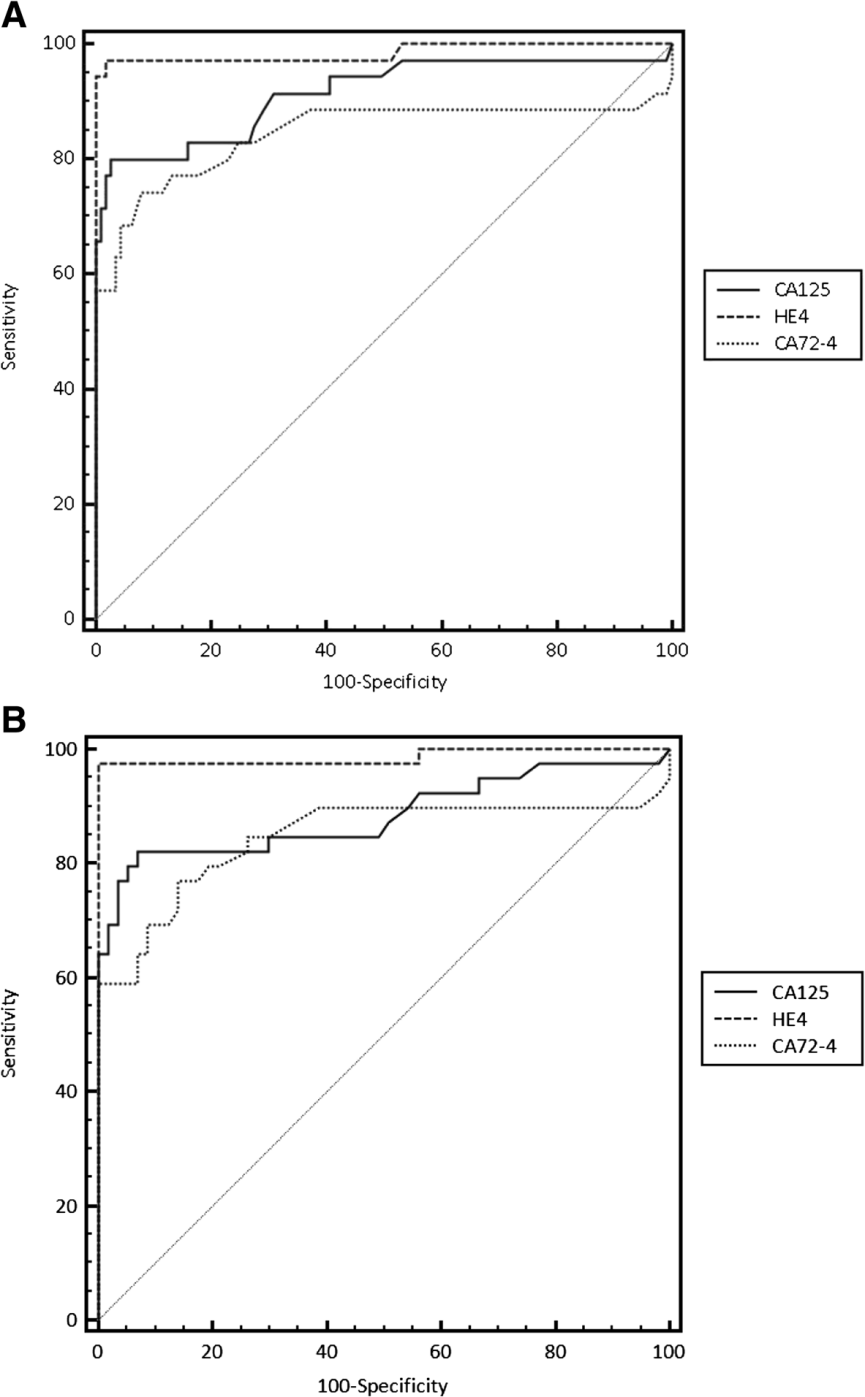
**ROC curves of healthy women, patients with benign mass and patients with endometriosis versus patients with ovarian cancer for CA125, HE4 and CA72-4. (A)** ROC curves of healthy women and patients with benign mass versus patients with ovarian cancer for CA125, HE4 and CA72-4. HE4 AUC = 0.985; CA125 AUC = 0.921; CA72-4 AUC = 0.843c The overall difference in AUCs between HE4 and CA72-4 was the only one statistically significant (P = 0.012). **(B)** ROC curves of patients with endometriosis versus patients with ovarian cancer for HE4, CA125 and CA72-4. HE4 AUC = 0.986; CA125 AUC = 0.883; CA72-4 AUC = 0.845.

Diagnostic performance of the markers in discriminating malignant from benign gynecologic conditions was verified using ROC analysis. The resultant accuracy (ROC Area) values for HE4, CA125 and CA72-4 and their corresponding ROC curves are shown in Figure 2A. All the three markers showed good performance, with AUCs of 0.985, 0.921 and 0.843 for HE4, CA125 and CA72-4 respectively. When the ROC analysis was performed for endometriosis, AUCs were 0.986 for HE4, 0.883 for CA125 and 0.845 for CA72-4 (Figure 2B).

## Discussion

Endometriosis is a known cause of CA125 elevation and represents a common gynecologic disorder in women of reproductive age [2]. Generally the diagnosis of ovarian endometriosis is made by clinical and imaging technique examinations [31] and confirmed by surgery with histological examination [6]. Recently surgical treatment of ovarian endometriosis in women desiring pregnancy has been criticized because of the risk of ovarian healthy tissue damage [13,14]. Therefore, in selected cases with ovarian endometrioma treated by medical therapy or undergoing assisted reproductive techniques (ART) without prior surgery, a correct diagnosis is mandatory. In these cases, the use of tumor markers with high sensitivity and specificity could help to reduce the risk, even small, of undetected ovarian cancer. In fact, there is a recognized association between endometriosis and clear cell, low-grade serous and endometrioid ovarian cancer [32]. So far, very little is known about the underlying factors involved in the malignant progression of endometriosis. For more than two decades CA125 has been the only marker employed in the diagnosis of EOC, but, although overexpressed in more than 80% of ovarian cancers, it lacks of specificity [33].

In the present study, we investigated the role of serum CA125, HE4 and CA72-4 in the diagnostic evaluation of ovarian endometrioma and adnexal mass. In agreement with data reported in literature [19], more than 50% of women with endometriosis expressed high levels of CA125, confirming the low specificity of this marker.

It was recently observed that HE4 rarely increases in benign gynecologic conditions suggesting its complementary role to CA125 [18,19,25,34,35]. In women with endometriosis, Moore et al. observed a marked difference between HE4 levels, which was increased only in 3% of cases, compared to CA125, which was elevated in 67% of cases [19]. Hamed et al. showed that serum HE4 and CA125 concentrations were significantly higher in patients with ovarian cancer compared with levels observed in patients with benign disease or healthy controls. In their study CA125 and HE4 had high sensitivity (90% *vs* 83.3%) and combining the two markers EOC were correctly detected in 97% of cases [36]. Moreover HE4 measurement, in healthy premenopausal women as well as in women with endometriosis, can be carried out at any phase of the menstrual cycle, and irrespective of hormonal therapy, extending the benefits of HE4 use in clinical practice [37,38]. However, HE4 overexpression has been also observed in non-oncologic conditions such as chronic kidney disease, which represents the most important known source of false-positive HE4 results [23]. In addition, recent studies have reported high HE4 levels also in some benign gynecological conditions such as uterine fibroma and pelvic inflammatory disease [8,19]. Moreover, HE4 levels in healthy women are affected by age, BMI and smoking [39].

In our study CA125 and HE4 yielded a sensitivity of 90% and 87% and a specificity of 70% and 100% respectively in the diagnosis of epithelial ovarian cancer. HE4 never increased in women with endometriosis and it was able to correctly discriminate malignant from all benign ovarian masses. Therefore we agree with previous reports that HE4 is the most useful marker for the differential diagnosis between EOC and ovarian endometriosis [19]. However, since some benign conditions can be associated with high HE4 levels, in selected cases CA72-4 may be useful for the differential diagnosis. Nevertheless the role of CA72-4 in the differential diagnosis between benign form malignant ovarian mass is still controversial [18].

In our study a slight and not statistically significant increase of CA72-4 was found in a small number of patients with endometriosis, with the highest observed value of 6.2 U/mL, which is indeed a borderline value, found only in one woman. Our data confirm the results reported by Lenhard et al. who showed that CA125 but not CA72-4 tends to be increased in the presence of endometriosis [24]. A multimarker approach, consisting of HE4, CA125 and CA72-4, can provide a more accurate tool for a differential diagnosis of patients with ovarian endometriotic cysts, other benign ovarian masses and ovarian cancer.

## Conclusions

In conclusion our results suggest that the use of serum HE4, CA125 and CA72-4 may be a valuable approach for distinguishing patients with ovarian endometrioma or other benign adnexal masses from those with ovarian malignancy. This approach could reduce medical costs related to more expensive diagnostic procedures and it may have a reassuring effect on the patient. Further studies are needed to confirm these results.

## Abbreviations

US: Ultrasound; CA125: Cancer antigen 125; EOC: Epithelial ovarian carcinoma; HE4: Human epididymis protein 4; CA72-4: Cancer antigen 72–4; TVUS: Transvaginal ultrasound; MRI: Magnetic resonance imaging; rASRM: revised American Society of Reproductive Medicine; FIGO: International Federation of Gynecology and Obstetrics; CLEIA: Chemiluminescent enzyme immunoassay; ALP: Alkaline phosphatase; AMPPD: 3-(2IGO: International Fedthoxy-4- (3(3zyme immunoassay; EIA: Immunoenzymatic assay; HRP: Horseradish peroxidase; PPV: Positive predictive values; NPV: Negative predictive values; ROC: Receiver operator characteristic; AUC: Areas under the curve; ART: Assisted reproductive techniques.

## Competing interests

All authors declare that they have no competing interest.

## Authors’ contributions

EA and MGP conceived and design the experiments. EA, MGP, RF, PS and AT performed the experiments. EA, MGP, TG, LF and PBP analyzed the data. TG contributed reagents/material/analysis tools. EA, MGP, RF, PS and AT wrote the paper. All authors read and approved the final manuscript.
